# Staphylococcal Protein A Promotes Colonization and Immune Evasion of the Epidemic Healthcare-Associated MRSA ST239

**DOI:** 10.3389/fmicb.2016.00951

**Published:** 2016-06-27

**Authors:** Xufen Hong, Juanxiu Qin, Tianming Li, Yingxin Dai, Yanan Wang, Qian Liu, Lei He, Huiying Lu, Qianqian Gao, Yong Lin, Min Li

**Affiliations:** ^1^Department of Laboratory Medicine, Huashan Hospital, Shanghai Medical College, Fudan UniversityShanghai, China; ^2^Department of Laboratory Medicine, Renji Hospital, School of Medicine, Shanghai Jiao Tong UniversityShanghai, China

**Keywords:** healthcare-associated methicillin-resistant *Staphylococcus aureus*, protein A, colonization, adhesion, immune evasion

## Abstract

The highly successful epidemic of healthcare-associated methicillin-resistant *Staphylococcus aureus* (HA-MRSA) ST239 is a growing concern worldwide, due to its progressive adaptation to the highly selective environment of the healthcare system. HA-MRSA ST239 display the reduced virulence and successfully colonize in hospital settings, while the emergent community-associated MRSA (CA-MRSA) maintain full virulence and cause infections in the community environment. Our aim was to investigate what enables *S. aureus* ST239 to be highly adaptive under hospital circumstances and gradually progress to a series of widespread invasive infections. We found that *spa* expression of HA-MRSA ST239 is much higher than that of CA-SA ST398. And we discovered that the highly production of staphylococcal protein A (SpA), having no concern with *spa* gene structure, enhances nasal colonization and cell adhesion in ST239. *S. aureus* ST239 defends against the adaptive immune response by resisting phagocytosis and inducing apoptosis of B cells through expression of surface-anchored and released protein A, facilitating its dissemination within the circulatory system to other organs. Protein A also plays another key role in subverting the host immune response through its ability to induce early shedding of TNF-α receptor 1 (TNFR1) from phagocytic cells. The increased levels of soluble TNFR1 present during experimental *S. aureus* ST239 infection may neutralize circulating TNF-α and impair the host inflammatory response. Protein A is also a virulence factor, as tested in our bacteremia model in mice, contributing to the durative tissue damage of abscess formation sites in ST239 infection. These functions of protein A eventually benefit to widespread infections of *S. aureus* ST239. We draw the conclusion that Staphylococcal Protein A may be a crucial determinant in the colonization and immune evasion of ST239 infections, contributing to persistent spread in the hospital settings. These results suggest that antibodies against protein A may provide insights into the development of novel treatments against *S. aureus*, especially HA-MRSA.

## Introduction

*Staphylococcus aureus* is a significant pathogen causing a series of infections, both in and out of the hospital settings. Under high antibiotic selective pressure, *S. aureus* has evolved its own tactics for survival and rapidly developed into a multidrug-resistant pathogen, especially methicillin-resistant *S. aureus* (MRSA). The overall number of invasive MRSA infections was estimated to be 80,461 within the United States alone in 2011 (Dantes et al., 2013). Notably, ~20% of the healthy human population also has continuous nasal cavity colonization with *S. aureus*, which constitutes a major risk for opportunistic infections (Weidenmaier et al., 2012).

The challenge of MRSA is an international health issue, and Asia is amongst the regions with the highest prevalence rates of healthcare-associated (HA)-MRSA and community-associated (CA)-MRSA in the world (Chen and Huang, 2014). MRSA prevalence in China has reached 50 to 70% of total *S. aureus* isolates (Liu et al., 2009; Xiao et al., 2011). Laboratory-based surveillance studies have shown that ST239-SCC*mec*III is the most common HA-MRSA clone in a number of geographically dispersed Chinese hospitals, with ST5-SCC*mec*II being the second most common (Li et al., 2013; Xiao et al., 2013). In contrast, the major strain of CA-MRSA is ST59-SCC*mec*IV in children (Geng et al., 2010) and there is also an international emerging spread of livestock-associated MRSA ST398 between humans and animals, and also between humans and humans (Huijsdens et al., 2006; van Belkum et al., 2008; Wang et al., 2015). In mainland China, the most prevalent CA-SA clone in adult skin and soft tissue infections is ST398, having no apparent association with animal contact (Zhao et al., 2012). The highly successful infiltration and adaptation into the hospital setting of MRSA ST239 is globally well known, with its earliest reports being in the late 1970s in the United Kingdom and the United States (Cookson and Phillips, 1988; Dubin et al., 1992). Since the 1990s, ST239 has also been the most dominant nosocomial MRSA clone in China (Aires de Sousa et al., 2003; Chen et al., 2010).

Recent studies have explored the global epidemiology of ST239 (Harris et al., 2010), and the contribution of other mobile genetic elements such as *sasX* to the spread and survival of this clone (Li et al., 2012). Given that there are many principle differences between CA-MRSA and HA-MRSA, including the typical patients and clinical infectious features (Nathwani et al., 2008), it is most likely that the mechanism behind the infections caused by HA-MRSA with attenuated virulence is distinct from CA-MRSA. While MRSA ST239 is the predominant HA-MRSA clone globally, because of its successful adaptation and survival within the hospital environment, the factors promoting adaptation and persistent spread in Chinese epidemic ST239 clone remain unknown. We discovered that the *spa* expression of HA-MRSA ST239 is much higher than that of CA-SA ST398. Staphylococcal Protein A (SpA), a surface-anchored conserved structural protein expressed by all *S. aureus* strains, has long been known to block opsonophagocytosis through its Fc ɤ-binding capacity in the presence of host antibody (Peterson et al., 1977), as well as to bind Fab regions of the B cell receptor (membrane-anchored IgM; Graille et al., 2000), crosslinking of V_H_3 clan IgM (Goodyear and Silverman, 2003) and inducing V_H_3-biased plasmablasts. The superantigen activity of SpA leads to immunodominance, limiting host responses to other *S. aureus* virulence factors (Pauli et al., 2014). SpA induces B-cell proliferation without cytokine stimuli and the proper T-cell help due to the anergic processes mediated by other superantigens, leading to SpA-activated B-cell deletion and anergy (Pozzi et al., 2015). SpA also induces early shedding of TNF-α receptor 1 (TNFR1) from phagocytic cells. The increased levels of soluble TNFR1 present during experimental *S. aureus* infections may neutralize circulating TNF-α and impair the host inflammatory response (Giai et al., 2013). Above all, SpA plays an important role in immune evasion in the pathogenesis of staphylococcal infections. Here, we confirmed that the higher expression of SpA contributes to the durable colonization and immune evasion in ST239 clone. Furthermore, SpA may serve as a significant factor in long-term adaptation and persistent spread of HA-MRSA ST239 clone within hospital settings.

## Materials and methods

### Ethics statement

All animal experiments were performed following the Guide for the Care and Use of Laboratory Animals of the Chinese Association for Laboratory Animal Sciences (CALAS) and approved by the ethics committee of Huashan Hospital, Fudan University School of Medicine, Shanghai, China. Human heparinized venous blood was taken from healthy individuals in accordance with a protocol approved by the ethics committee of Huashan Hospital, Fudan University School of Medicine, Shanghai, China. All individuals gave written informed consent prior to donating blood.

### Bacteria and growth conditions

Bacteria were identified as staphylococci by classic microbiological methods including Gram staining and catalase and coagulase activity in rabbit plasma. *S. aureus* strains were further categorized by VITEK2 automated systems (BioMérieux, France). Community-associated *S. aureus* was defined as an isolate that was obtained either from an outpatient or from an inpatient ≤ 24 h after hospital admission and lacking the following risk factors: contact with the hospital environment in the 6 months preceding the culture, *S. aureus* infection history or residence in a long-term care facility in the 12 months preceding the culture, presence of a central vascular catheter at the time of infection, recent antibiotic use, injection drug use; healthcare-associated *S. aureus* was defined as an isolate that was obtained from an inpatient >24 h after hospital admission or having at least one of those risk factors. *S. aureus* strains used for RT-PCR and Western-blot of SpA expression were randomly selected from the clinical isolates with typically infectious manifestations at Shanghai teaching hospitals in the year of 2005–2012. The MRSA clinical isolate Ji99 (HA-MRSA ST239 strain), which used for the development of the *spa* mutant, was recovered from the sputum of an inpatient with pneumonia. The isolate 1059 (the CA-SA ST398 strain) was recovered from the skin abscess of an outpatient at Shanghai Hospital, China. The two strains were used for the following studies, due to their expression of *spa* were in the average of both RNA and protein level. Isolates were grown in tryptic soy broth (TSB; Oxoid, Basingstoke, Hampshire, UK) at 37°C with agitation and used in all animal work. *Escherichia coli* TOP10 was grown in Luria-Bertani broth (LB; Oxoid). When necessary, media were supplemented with ampicillin (100 μg/ml) for *E. coli* or chloramphenicol (10 μg/ml) for *S. aureus*.

### Antibiotic resistance tests

Antimicrobial susceptibility profiles of *S. aureus* isolates were determined by the agar dilution method on Mueller-Hinton agar, according to the recommendations and definitions from the Clinical and Laboratory Standards Institute (CLSI). Antimicrobial agents tested were purchased from Oxoid and included gentamicin, penicillin, cefoxitin cefazolin, levofloxacin, erythromycin, clindamycin, trimethoprim/sulfamethoxazole (TMP/SMX), fosfomycin, rifampicin, teicoplanin, linezolid, and vancomycin. ATCC29213 (*S. aureus*) and ATCC29212 (*Enterococcus faecalis*) were used as quality controls.

### Multi-locus sequence typing (MLST)

MLST was performed as previously described (Maiden et al., 1998). PCR amplicons of seven *S. aureus* housekeeping genes (*arcC, aroE, glpF, gmk, pta, tpi*, and *yqiL*) were obtained from chromosomal DNA. The sequences of the PCR products were compared with the existing sequences available at the MLST website (http://www.mlst.net), and the allelic number was determined for each sequence.

### *Spa* typing

Amplification and sequencing of the polymorphic X region of the *spa* gene were performed as described (Koreen et al., 2004). The *spa* type was assigned using the *S. aureus spa* type database (http://www.ridom.de/spaserver/).

### Allelic gene replacement by homologous recombination and genetic complementation

*S. aureus* ST239 (Ji99)/ST398 (1059) and variants with *spa* deletion were generated by allelic replacement using the pKOR1 shuttle vector (Bae and Schneewind, 2006). Briefly, 1 kb flanking regions up and downstream of *spa* were amplified, ligated, and inserted into pKOR1 using the BP Clonase II kit (Invitrogen, Life Technologies, Carlsbad, CA, USA; Bae and Schneewind, 2006). Recombinant plasmids were transduced with Φ85 into *S. aureus* ST239 (Ji99)/ST398 (1059) for integration into the chromosome and selection of deletion mutants (Supplementary Table 1). For *spa* complementation, DNA fragments were amplified with the primer pairs *spa*-Sma1-F/*spa*-BamH1-R (Supplementary Table 2), using genomic DNA of ST239 (Ji99) and ST398 (1059) as a template, respectively. The amplified fragments were digested with *Sma*1 and *BamH*1 and inserted into pCL55, circularized with T4 ligase and then transformed into *E*. *coli* DH5α. Once verified, all plasmids were given electroporation into *S*. *aureus* strain RN4220 and subsequently transduced into the *spa*-deletion mutant of *S*. *aureus* strain Ji99 (ST239Δ*spa*) with Φ85 to generate the complementation: Ji99Δ*spa* (p239), Ji99Δ*spa* (p398) and Ji99Δ*spa* (pCL55).

### Nasal colonization model

Female BALB/c mice were used for the nasal colonization model (eight mice per group). All mice were 6–8 weeks of age at the time of use and received drinking water containing ampicillin (100 μg/ml). *S. aureus* strains were grown to the mid-exponential growth phase, washed and resuspended in sterile PBS at 1 × 10^10^ CFUs per ml. Mice were anesthetized with isoflurane. The inoculum, which contained 1 × 10^8^ CFUs in 10 μl of PBS or PBS alone, was pipetted slowly into the nares of the anesthetized mice without touching the nose with the pipette tip. Three days after inoculation, eight mice per group were killed and evaluated for nasal carriage of *S. aureus*. The nasal region was wiped externally with 70% ethanol, and nasal tissue homogenized in 0.5 ml TSB. The total number of *S. aureus* CFUs per nose was assessed by plating 100 μl diluted nasal suspensions on TSB agar containing ampicillin (100 μg/ml; Li et al., 2012).

### RT-PCR and western blot of SpA expression

For RNA isolation, overnight cultures were diluted 1:100 in 50 ml TSB and incubated at 37°C with shaking at 220 rpm for 8 h. Complementary DNA (cDNA) was synthesized from total RNA using the QuantiTect reverse transcription system (Qiagen, Hilden, Germany) according to the manufacturer's instructions. Oligonucleotide primers were designed using Primer Express (Supplementary Table 2). The resulting cDNA and negative control samples were amplified using the QuantiTect SYBR green PCR kit (Qiagen). Reactions were performed in a MicroAmp Optical 96-well reaction plate using a 7500 Sequence Detector (Applied Biosystems, Foster City, CA, USA). Standard curves were determined for each gene, using purified chromosomal DNA at concentrations of 0.005–50 ng/ml. All quantitative qRT-PCR experiments were performed in duplicate, with *gyrB* as an internal control (Li et al., 2012).

*S. aureus* strains were inoculated from frozen stocks into TSB and grown overnight. The culture was diluted 1:100 and incubated at 37°C to post-exponential phase (8 h). Samples were removed from cultures according to the OD_600_ reading (OD_600_ = 2) and centrifuged at 20,000 × g for 1 min. The cell pellet was washed with 1 mL PBS solution and suspended in 20 μL TSM [50 mM Tris·HCl (pH 7.5), 0.5 M sucrose, 10 mM MgCl_2_], and placed at 37°C for 30 min to digest the cell wall envelope with lysostaphin (100 μg·mL^−1^). The digest was centrifuged at 20,000 × g for 1 min and the supernatant was taken as the cell wall proteins. The proteins released to culture were collected in supernatants and added to 1/5 volume of 100% TCA. Samples were vortexed and incubated at −20°C for 30 min. Precipitated proteins were collected by centrifugation at 22,000 × g for 15 min. The supernatant was discarded, and protein pellets were washed twice with ice-cold acetone, air-dried, and solubilized in 25 μL 4% SDS in 0.5 M Tris·HCl (pH 8.0), collected as the released proteins in culture. An equal volume of SDS-PAGE sample buffer [125 mM Tris·HCl (pH 6.8), 4% SDS, 20% glycerol, 10% 2-mercaptoethanol, 0.01% bromophenol blue] was added, and protein samples were boiled for 10 min. Proteins were separated on 12% SDS-PAGE gels and electrotransferred to a PVDF membrane. The membrane was treated in blocking buffer (5% milk powder in PBST), followed with rabbit anti-SpA (1:1000), then incubated at room temperature and anti-rabbit immunoglobulin G (IgG) HRP-conjugated secondary antibody (1:2000, Cell Signaling, Technology, Danvers, MA, USA) added. The reactions were made visible using diaminobenzidine. To ensure consistency between blots, SrtA was used as fractionation control. Densitometry analysis was performed with ImageJ software for each protein band in reference to the corresponding sortase A band.

### *S. aureus* infection of keratinocytes and epithelial cells

For adhension assay, *S. aureus* was cultured in TSB and cell pellet was washed twice with RPMI1640 or F12K medium (Kaighn's Modification of Ham's F-12 Medium). Human immortal keratinocytes cells HaCaT were cultured in RPMI 1640 medium supplemented with fetal bovine serum (FBS, 10%) in T75 flasks at 37°C and 5% CO_2_. Cells were liberated from flasks using trypsin-EDTA solution (Sigma-Aldrich, St Louis, MO, USA), resuspended in culture medium and seeded at 2 × 10^6^ cells/well, at a multiplicity of infection (MOI) of 100 in a final volume of 500 ul for 120 min at 37°C with 5% CO_2_. Cells were washed 3 times in RPMI 1640, and 10^8^ CFU *S. aureus* were added and incubated with the cells for 120 min. Coverslips that were used to determine the total number of associated CFU (adherent and internalized) were dip washed three times and subsequently lysed by the addition of 500 μl 0.1% deoxysodium cholate solution. Bacterial CFU were enumerated by serial dilutions of epithelial cell lysates and plating onto TSA plates. The procedure of *S. aureus* infection of epithelial cells A549 was the same as described above, except that cells were cultured in F12K medium supplemented with fetal bovine serum (FBS, 10%) and L-glutamine (2 mM).

### Phagocytosis and survival of bacteria in whole blood

*S. aureus* strains were grown to mid-exponential growth phase, washed, and resuspended in sterile PBS at 10^8^ CFU/ml. 10^7^ CFU of *S. aureus* in 100 μl PBS were pipetted slowly into 10 ml heparinized blood, mixed gently for 30 s and incubated at 37°C. Blood smears from each time point (0, 15, 30, 45, 60, and 90 min) were prepared and cells were stained with a modified Wright-Giemsa stain. The total number of *S. aureus* (bound, ingested and those within 10 μm distance of neutrophils) was evaluated using 50 neutrophils per assay. As the clinical strains of ST239 are highly resistant to gentamycin (Li et al., 2013), surviving still in the existent of 100 μg/ml gentamicin, calculation of phagocytosed bacteria was not performed in gentamycin protection assay. Percent phagocytosis was calculated using the equation: number of ingested bacteria/∑ (PMN-associated bacteria, PMN-bound bacteria, ingested bacteria) × 100. To determine survival rates, 100 μl of heparinized blood was plated on TSA containing 5% sheep blood for *S. aureus* detection (Li et al., 2012).

### Infection of RAW 264.7 cells with *S. aureus* and detection of soluble TNFR1

RAW 264.7 cells (a mouse macrophage cell line) were cultured in RPMI 1640 medium (Life Technologies, Grand Island, NY, USA) supplemented with 10% FBS, 0.11 mg/ml pyruvate (Sigma-Aldrich), 0.29 mg/ml GlutaMAX (Life Technologies), and 1 × nonessential amino acids (Life Technologies). RAW 264.7 cells cultured to confluence were weaned from serum 24 h before exposure to stimuli. *S. aureus* strains were grown in TSB and suspended in RPMI 1640 medium at a concentration of 1 × 10^8^ CFU/ml. RAW 264.7 cells were added to prepared *S. aureus* strains and centrifuged at 1000 × g for 5 min before incubation in 5% CO_2_ at 37°C. The supernatants were collected and used to detect soluble TNFR1 at 30, 60, and 120 min after infection (Giai et al., 2013). Soluble TNFR1 was quantified in culture supernatants by enzyme-linked immunosorbent assays (ELISA) using DuoSet antibody pairs (Abcam, Cambridge, MA, USA).

### Bacteriemia of *S. aureus* in mouse sepsis model

Overnight cultures of 10 clinical isolates *S. aureus* ST239 and ST398 were diluted 1:100 into fresh TSB and grown for 4 h at 37°C. Staphylococci were pelleted, washed, and suspended in PBS to OD_600_ 4 (~1 × 10^9^ CFU/ml). Inocula were quantified by spreading sample aliquots on TSA and enumerating colony formation. BALB/c mice (4–6 week old, female) were anesthetized via inhalation with isoflurane (100 mg/ml per kilogram of body weight). Mice were infected with 100 μl of bacterial suspension (1 × 10^8^ CFU) by retroorbital injection and monitored for survival.

### Renal abscess model

Overnight cultures of *S. aureus* ST239-Ji99 and its isogenic mutant were diluted 1:100 into fresh TSB and grown for 4 h at 37°C. Staphylococci were pelleted, washed, and suspended in PBS to OD_600_ 0.4 (~1 × 10^8^ CFU/ml). Mice were sublethal challenged with 100 μl of bacterial suspension (1 × 10^7^ CFU). Animals were euthanized on day 7 and 15 post-infection. Both kidneys were removed, and the staphylococcal loads in the kidney were analyzed after homogenizing renal tissue with PBS and 0.1% Triton X-100. Serial dilutions of homogenate were spread on TSA and incubated for colony formation. The left kidney was examined by histopathology. Briefly, kidneys were fixed in 10% formalin for 24 h at room temperature. Tissues were then embedded in paraffin, thin-sectioned (4 μm), stained with hematoxylin and eosin, and inspected by light microscopy to observe abscess lesions. Abscess lesions were identified as foci of infiltrated immune cells (mainly neutrophils) and/or bacterial communities (staphylococcal abscess community; Kim et al., 2011).

### Statistical analysis

Unpaired two-tailed Student's *t*-tests were performed to analyze the statistical significance of nasal colonization, *spa* expression, bacterial adhesion, blood survival data, ELISA data, survival rates and bacterial loads in the experimental animal infection models. All data were analyzed using Prism (GraphPad Software, Inc., La Jolla, CA, USA), and *P* < 0.05 were deemed statistically significant.

## Results

### The nasal colonization rate of clinical HA-MRSA ST239 isoaltes is much higher than CA-SA ST398 isoaltes

**Figure 1:**
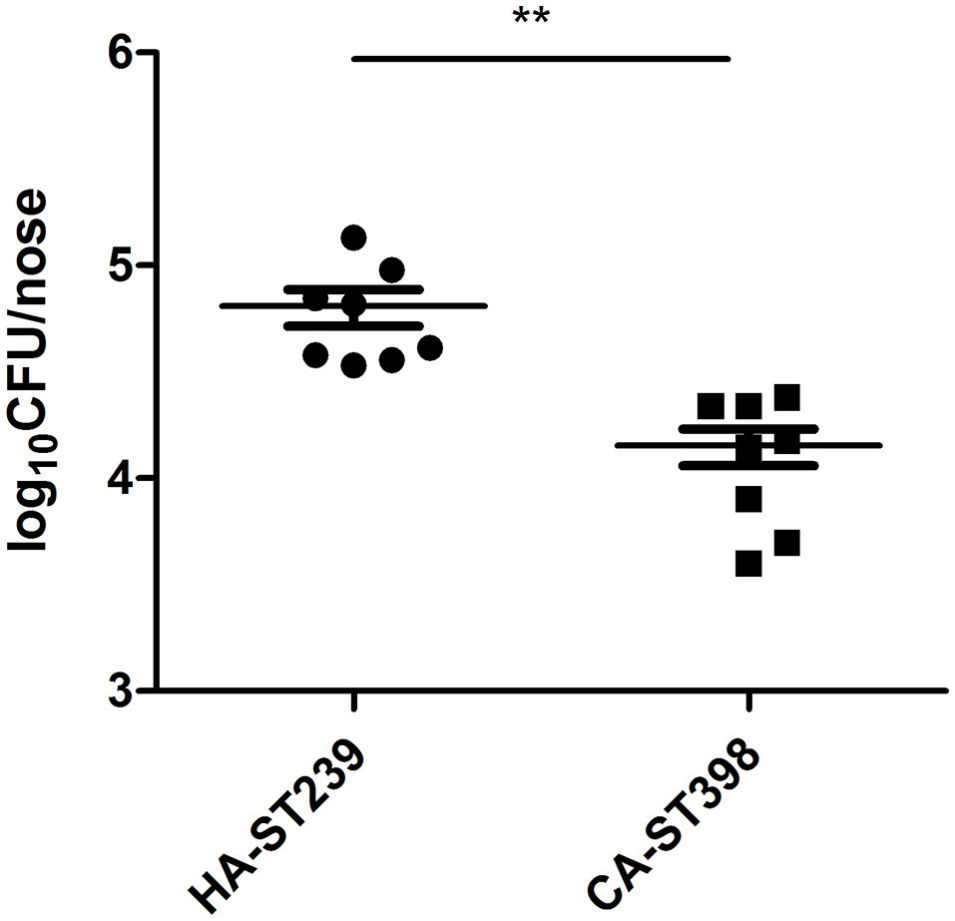
**The nasal colonization rate of clinical HA-MRSA ST239 isoaltes is much higher than CA-SA ST398 isoaltes Mice were inoculated intranasally with clinically isolated HA-MRSA ST239 and CA-SA ST398 isolates (1 × 10^**8**^ CFU per mouse)**. After 3 days, mice were euthanized and the nasal bacterial burden established. Inoculation with the HA-MRSA ST239 isolates resulted in significantly more colonization than the CA-SA ST398 isolates. Statistical analysis was performed using a nonparametric *t*-test (*n* = 8 per group), ^**^p < 0.01.

Colonization of the nares with MRSA, either present at admission to the hospital or acquired during hospitalization, has been proven to be a risk factor for subsequent MRSA infections (Davis et al., 2004; Wertheim et al., 2004). ST239 is a worldwide prevalent healthcare-acquired MRSA, and is especially dominant in China (Li et al., 2013; Xiao et al., 2013). To explore the nasal colonization ability of ST239, we established nasal colonization in specific pathogen free BALB/c mice with randomly selected clinical ST239 and ST398 strains. Prior to inoculation, mice were administered ampicillin (all strains were ampicillin-resistant) in their drinking water for 24 h and for the duration of the experiment in order to reduce interference from commensal bacterial flora. On day 3 after inoculation, nasal tissue was excised and homogenized and the number of CFU per nose enumerated. It was observed that there was a significant difference in the colonized number of ST239 compared with ST398 (*p* = 0.0091), suggesting that the nasal colonization of ST239 was much higher than ST398 (Figure 1). This was in accordance with the persistent spread caused by ST239. However, the factor promoting the colonization of ST239 MRSA remained to be determined.

### *spa* expression is elevated in clinical HA-MRSA ST239 isolates, both on the RNA and protein level, compared with CA-SA ST398 isolates

**Figure 2:**
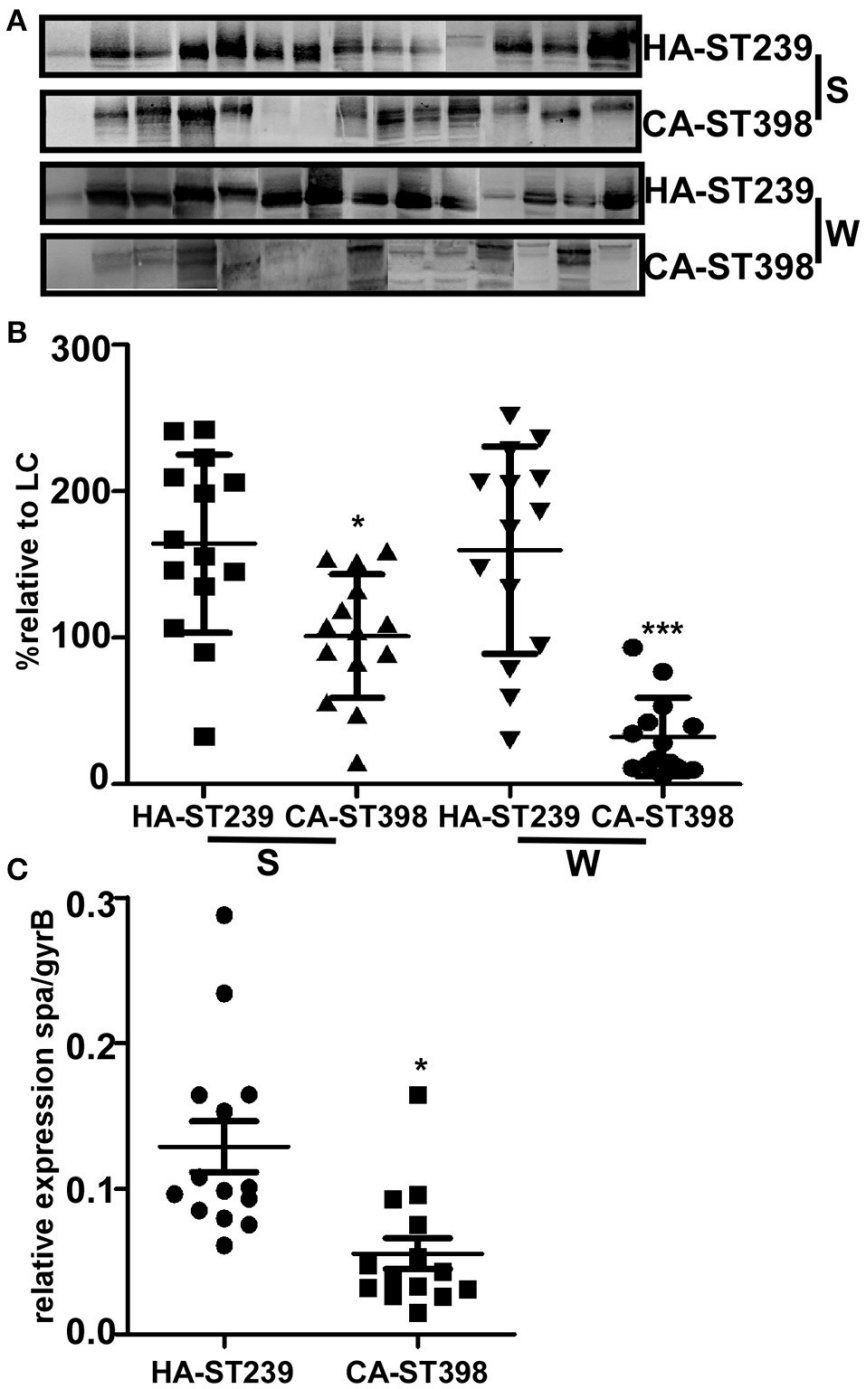
*****spa*** expression is elevated in clinical HA-MRSA ST239 isolates, both on the RNA and protein level, compared with CA-SA ST398 isolates**. **(A)** Clinical HA-MRSA ST239 and CA-SA ST398 isolates were cultured for 8 h then centrifuged to separate the extracellular protein A (marked as “S”) from the bacterial cell wall-associated protein A (marked as “W”). After treatment of the staphylococcal cell wall envelope with lysostaphin, proteins in both fractions were analyzed by immunoblotting with polyclonal antibodies against protein A. The original gels are provided in the Supplementary Figure 1. **(B)** Semiquantitative densitometry analysis of each protein band in reference to the corresponding sortase A band was performed using ImageJ software. **(C)** RNA transcripts were quantified in reference to the transcription of the housekeeping gene gyrB at 8 h (post-exponential phase) cultures of clinical HA-MRSA ST239 and CA-SA ST398 isolates. Each dot represents a different strain. Statistically significant differences between ST239 and ST398 are indicated: ^*^p < 0.05, ^***^p < 0.001

*spa* expression was analyzed using quantitative real-time RT-PCR on a series of clinically isolated ST239 and ST398 strains with typical infectious manifestations at Shanghai teaching hospitals in the year of 2005–2012, 14 isolates, respectively. After 8 h of culture, the transcription level of *spa* in HA-MRSA ST239 isolates was higher than that of CA-SA ST398 isolates (Figure 2C). We then investigated the protein expression level by western blot. SpA is both cell wall-anchored and partially secreted into the supernatant (Becker et al., 2014). Notably, the two components were both detected. We found that a large proportion of HA-MRSA ST239 strains expressed more SpA compared with CA-SA ST398 in both cell wall and secreted fragments (Figures 2A,B), indicating that overall HA-MRSA ST239 strains expressed more SpA, which may contribute to the persistent colonization with HA-MRSA ST239 strains.

### SpA improves the nasal colonization ability of *S. aureus* ST239

**Figure 3:**
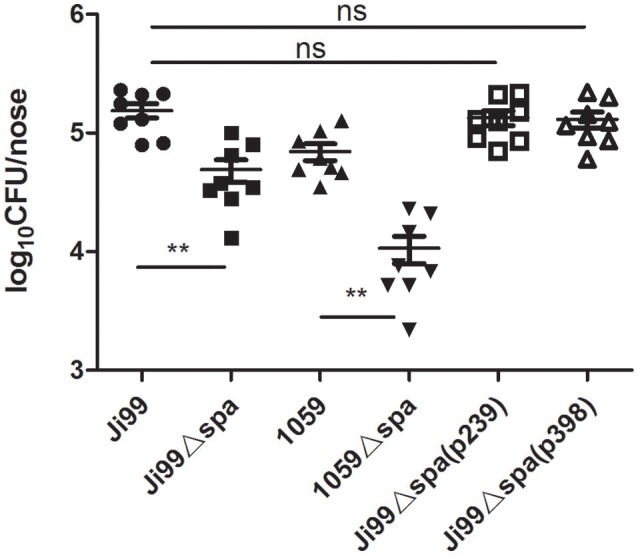
**SpA improves the nasal colonization ability of ***S. aureus*** ST239**. Mice were inoculated intranasally with WT ST239-Ji99, ST398-1059, their Δ*spa* mutant strains (Ji99Δ*spa*, 1059Δ*spa*) and complemented strains [Ji99Δ*spa*(p239), Ji99Δ*spa*(p398)] (1 × 10^8^ CFU per mouse). After 3 days, mice were euthanized and the nasal bacterial burden determined. Statistical analysis was performed using a nonparametric *t*-test (*n* = 8 per group), ^**^p < 0.01.

The human nose is the primary reservoir of *S. aureus*. Several molecular factors of *S. aureus* have been identified as being potentially responsible for nasal colonization, including teichoic acids (Weidenmaier et al., 2004), specific surface proteins (Wertheim et al., 2008; Corrigan et al., 2009), and immune-modulatory factors (Jin et al., 2004; Clarke et al., 2007). SpA is also an important factor blocking complement activation and opsonization (Rooijakkers et al., 2005), which is detectable at the RNA level in nasal swabs (Burian et al., 2010). This prompted us to investigate the relationship between SpA and nasal colonization. We constructed the Δ*spa* mutant strains of ST239-Ji99 and ST398-1059. Additionally, the *spa* gene of Ji99 [Ji99Δ*spa*(p239)] and 1059 [Ji99Δ*spa*(p398)] was complemented into the Ji99Δ*spa* mutant strain, and established nasal colonization models with wild type, the Δ*spa* mutant and the complemented strains. As depicted in Figure 3, colonization of the nasal cavity was dramatically decreased with both Δ*spa* mutant strains compared with the wild type strains, complementation of either Ji99 *spa* or 1059 *spa* reversed the nasal colonization of wild type ST239. Additionally, there was also a statistical difference in the two *spa* deletion strains (Figure 3), indicating that SpA is not the only factor affecting colonization between ST239 and ST398.

### SpA improves the keratinocyte and epithelial cell adhesion ability of *S. aureus* ST239

**Figure 4:**
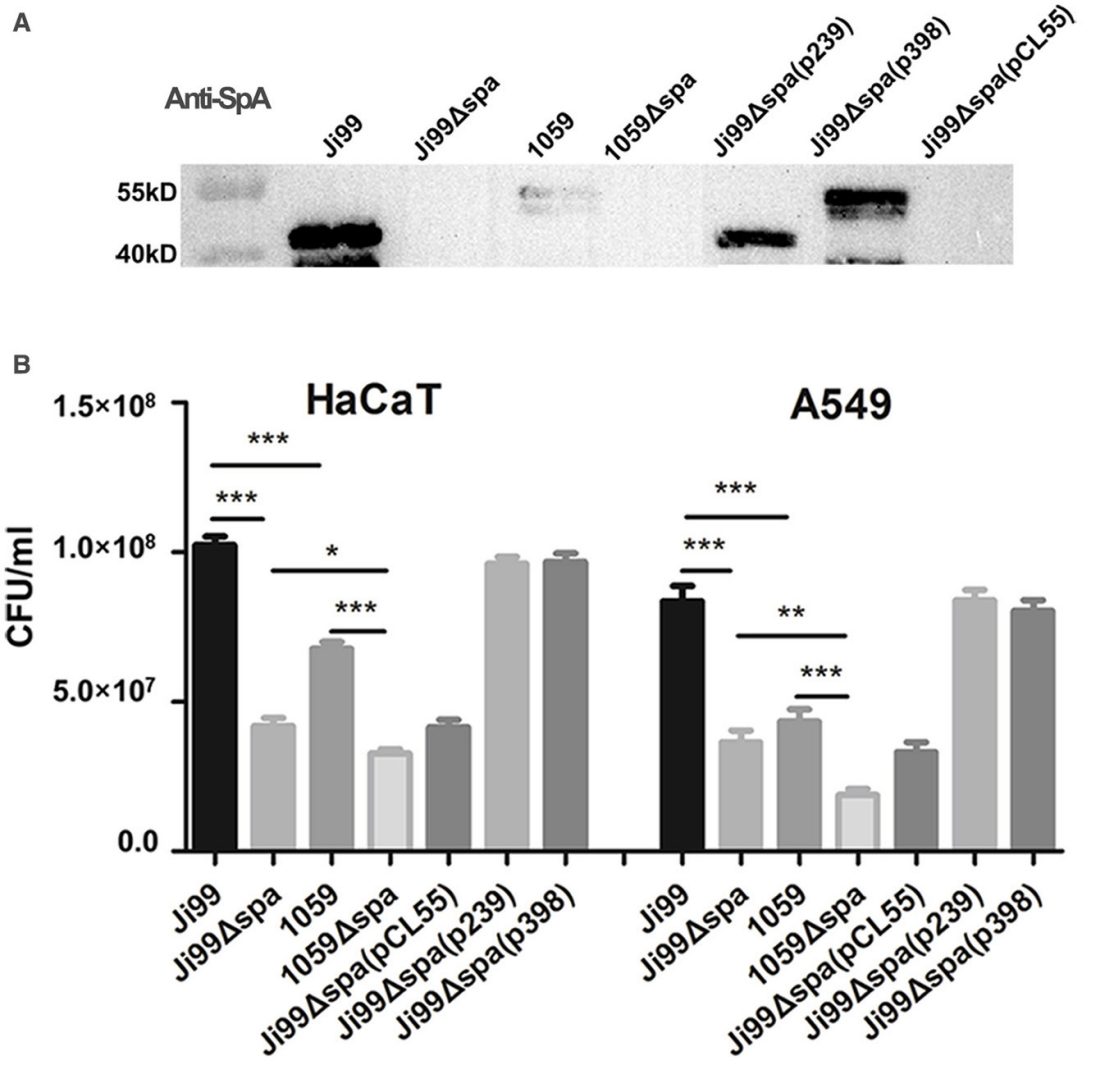
**SpA improves the keratinocyte and epithelial cell adhesion ability of ***S. aureus*** ST239. (A)** Anti-SpA western-blot of WT bacteria, Δ*spa* mutant, Δ*spa* mutant complemented with the ST239 *spa* gene [Δ*spa*(p239)], ST398 *spa* gene [Δ*spa*(p398)], or vector [Δ*spa*(pCL55)]. **(B)** Colony counts of adhesive and internalized bacteria above in HaCaT keratinocytes and A549 epithelial cells infected for 2 h. An ~0.25-fold increase could be seen for ST239 compared with ST398 and ~1-fold increase compared with the Δ*spa* mutant. Statistical differences were determined by one-way ANOVA with Tukey's multiple-comparison test, ^*^p < 0.05, ^**^p < 0.01, ^***^p < 0.001. Data are representative of three independent experiments.

As SpA is a surface-associated protein within the microbial surface components recognizing adhesive matrix molecules (MSCRAMMs) family (Speziale et al., 2009), we wondered if this protein also played a role in the interaction with keratinocytes and epithelial cells. The *spa* mutant (Ji99Δ*spa*, 1059Δ*spa*) and complemented strains [Ji99Δ*spa*(p239), Ji99Δ*spa*(p398)] were constructed, with the pCL55 vector complement [Ji99Δ*spa*(pCL55)] being used as a control. The expression of SpA in the complement strains were verified by western-blot (Figure 4A). All strains were added to cultured human keratinocytes (HaCaT) and lung epithelial cell (A549) monolayers for 2 h to measure the attachment of bacteria. As expected, the number of attached and internalized wild type Ji99 bacteria was greater than the number of 1059 bacteria, and both were markedly decreased in the *spa* deletion strains. The complement strains of SpA from either ST239(Ji99) or ST398(1059) could reverse the bacteria adhension ability (Figure 4B). It was likely that the difference between the two clinical strains had little connection with the different *spa* gene structures. These results suggested that protein A enhanced the keratinocytes and epithelial cells adhesion of HA-MRSA ST239 strains and promoted the colonization in return. The statistical difference in the two *spa* deletion strains indicated that SpA was not the only factor affecting the bacteria adhesion (Figure 4B).

### Resistance to phagocytosis is decreased in the ST239 Δ*spa* strain

**Figure 5:**
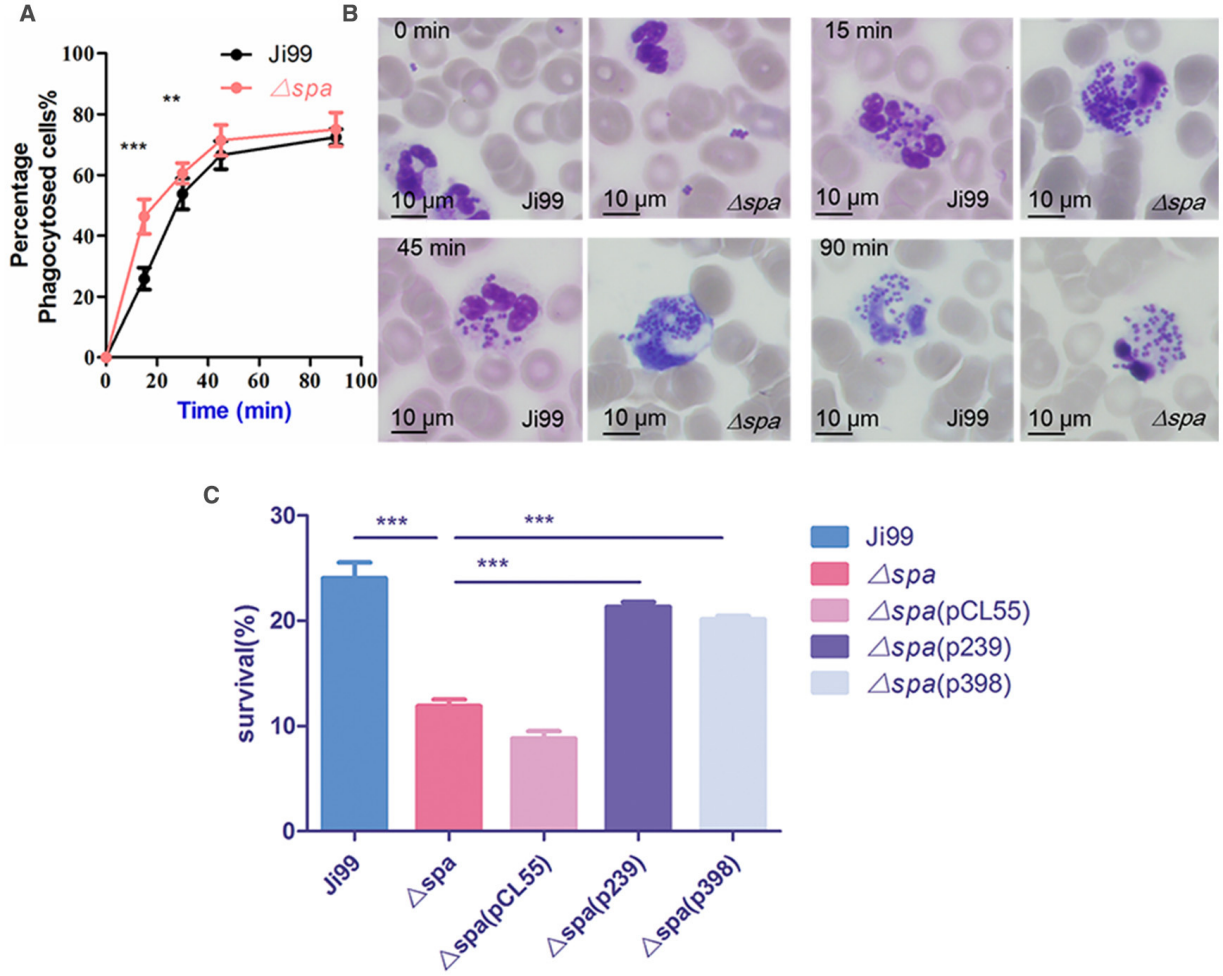
**Resistance to phagocytosis mediated by protein A is enhanced in the HA-MRSA ST239 strains**. **(A)** Phagocytosis assay. Phagocytosis by human neutrophils of ST239 or the Δ*spa* mutant bacteria was examined by light microscopy. Significant differences were seen at 15 and 30 min incubation. **(B)** Microscopic examination of phagocytosis. Slides were stained using a modified Wright-Giemsa stain at 0, 15, 45, and 90 min. There were fewer phagocytized *S. aureus* ST239 bacteria in neutrophils compared with the mutant strain from 15–45 min. **(C)** Survival in human blood at *t* = 15 min (Other time such as 30 and 45 min time points are statistically significant, but not that much compared to the 15 min time point). Statistically significant differences are indicated, ^**^p < 0.01, ^***^p < 0.001.

Neutrophils play a central part in protecting humans against *S. aureus* infection. Staphylococcal entry and replication in host tissues lead to the release of bacterial products that produce inflammatory signals. Neutrophils answer this call, extravasating from blood vessels, migrating toward the site of infection to phagocytose and kill bacteria or to immobilize and damage the pathogen (Thammavongsa et al., 2015). When confronted with neutrophils, most bacteria are more easily phagocytized in comparison with *S. aureus*. The liberated SpA together with the wall-anchored could interact with Fcɤ region of IgG (Peterson et al., 1977) via the IgG-binding domains (Deisenhofer, 1981), coating the surface of the cell with IgG molecules unable to be recognized by the neutrophil Fc receptor and blocking the neutrophil's phagocytosis of *S. aureus*. Furthermore, protein A-rich strains are phagocytized more slowly than strains with low expression of protein A (Peterson et al., 1977). Therefore, we investigated the antiphagocytic effect of protein A in WT ST239-Ji99 with high levels of protein A and the Δ*spa* mutant strain with no protein A. After 15 min of incubation with normal human whole blood, the Δ*spa* mutant strain underwent a greater degree of phagocytosis than WT (*p* < 0.0001). This effect could also be seen at 30 min of incubation (*p* = 0.0031), as seen in the blood smear from 15–45 min after phagocytosis (Figures 5A,B). The complementation of Ji99 *spa* gene and 1059 *spa* gene resulted in the same survival rate in human blood as the Ji99 WT after 15 min of phagocytosis, which was much higher than the Δ*spa* mutant (Figure 5C). The higher anti-phagocytosis capacity of ST239, largely due to its high expression of protein A, increased its immune evasion from host immune cells.

### Protein A attenuates the inflammatory response caused by ST239 through early shedding of TNFR1

Evading the host immune response is a vital strategy to enable duration in *S. aureus* infection. Protein A plays another key role in subverting the host immune response through its ability to induce early shedding of TNF-α receptor 1 (TNFR1). The increased levels of soluble TNFR1 present during experimental *S. aureus* infection may neutralize circulating TNF-α and impair the host inflammatory response (Giai et al., 2013). Among the proinflammatory cytokines induced, TNF-α has been shown to be crucial for the eradication of bacteria in other experimental models (Nakane et al., 1995). As for our clinical ST239 isolates with high expression of protein A, the specific mechanism involved remained to be elucidated. We hypothesized the lower virulence of ST239 may be due to the higher expression of SpA, which could attenuate the inflammatory response. Therefore, the kinetics of TNFR1 shedding in response to whole live clinical isolates of *S. aureus* ST239 and ST398 was investigated in macrophages.

**Figure 6:**
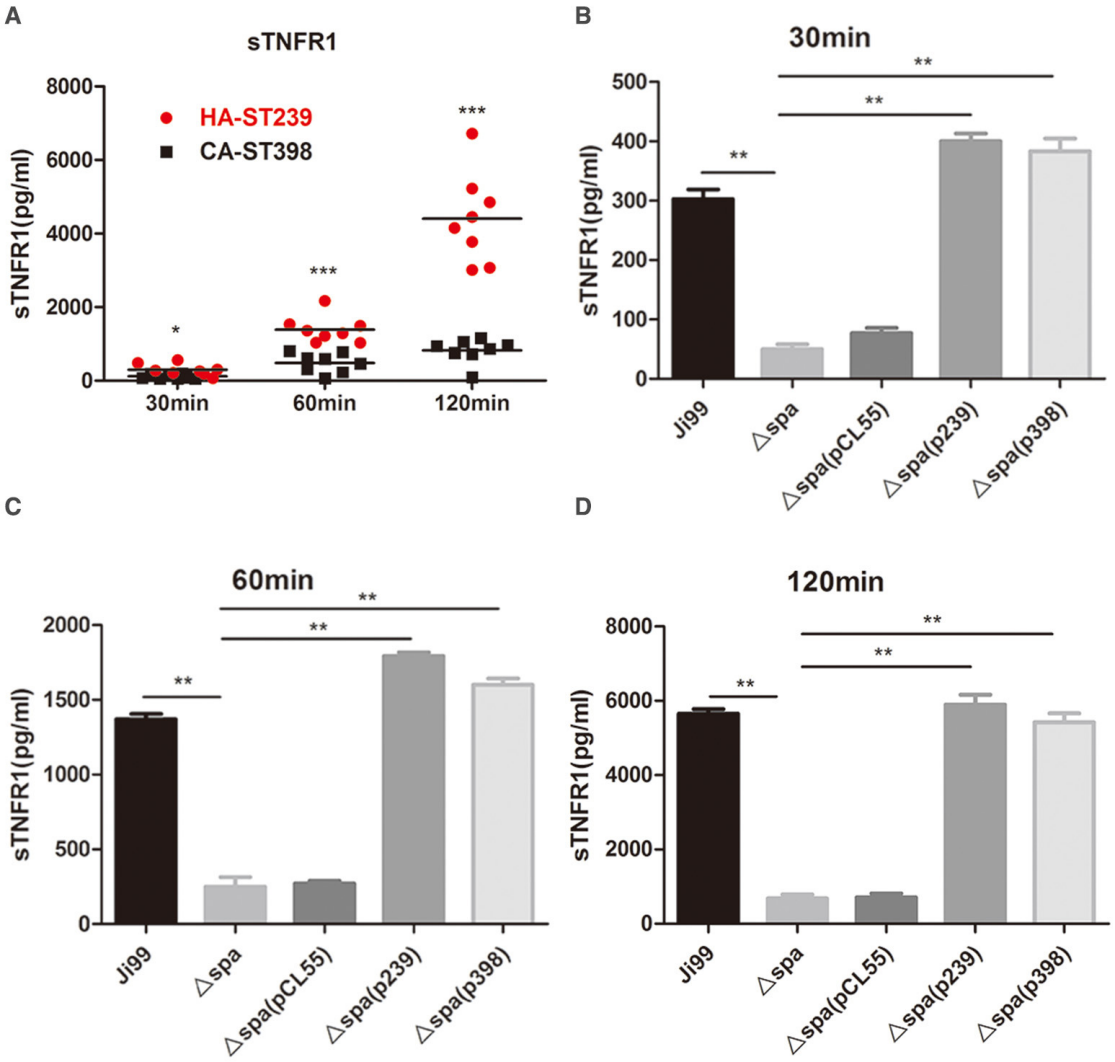
**Protein A induces early shedding of TNFR1, attenuating the inflammatory response after infection with HA-MRSA ST239**. RAW 264.7 cells were stimulated with the clinical isolates HA-MRSA ST239, CA-SA ST398 **(A)** or wild-type ST239-Ji99, the Δ*spa* mutant and complemented strains **(B–D)** for the times indicated. The concentration of sTNFR1 was determined by ELISA. Data are means and standard deviations of cumulative data from three independent experiments (*n* = 3 for each experiment). *Asterisks* indicate significant differences (^**^p < 0.01) by a nonparametric *t*-test.

Shedding of TNFR1, as well as an obvious distinction between ST239 and ST398 strains, occurred as soon as 30 min after exposure of macrophages to *S. aureus*. The difference in shedding was even larger at 120 min exposure (Figure 6A). With the Δ*spa* mutant, there was much less TNFR1 shedding compared to WT ST239-Ji99. When the *spa* gene was complemented into the mutant, either Ji99 *spa* or 1059 *spa*, TNFR1 shedding returned to the WT level from 30 to 120 min after infection (Figures 6B–D). Thus, the TNFR1 shedding effect of protein A was greater in ST239, constituting a crucial step in immune evasion of *S. aureus* infections.

### *SpA* may contribute to the durative damage in host infected with HA-MRSA ST239

**Figure 7:**
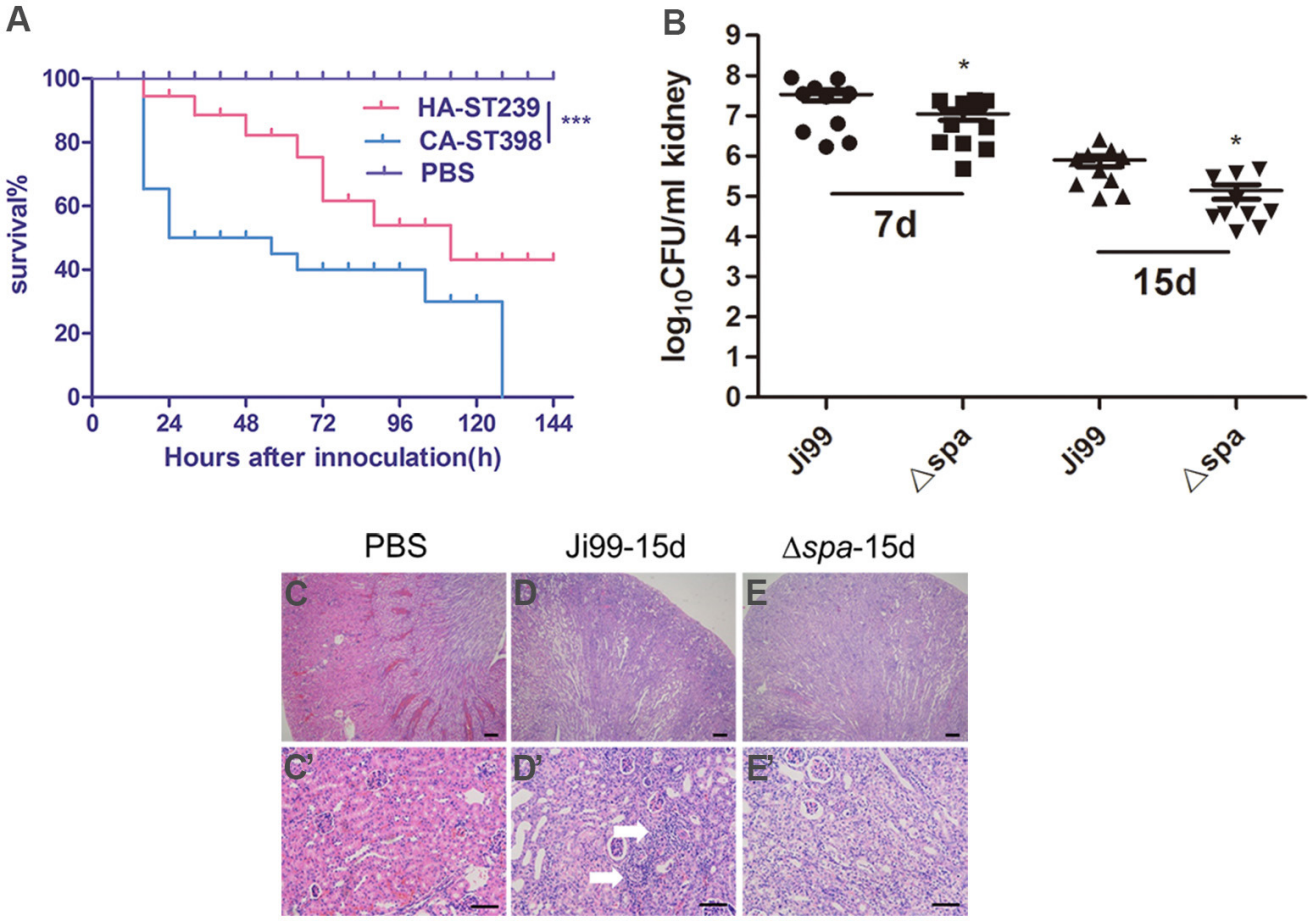
**SpA contributes to the durative damage in host infected with HA-MRSA ST239**. **(A)** Percentage of surviving mice in mice sepsis model challenged with 1 × 10^9^ CFU of clinically isolated ST239 and ST398. The mortality over the experimental period was significantly lower for mice infected with HA-MRSA ST239 compared to those infected with CA-SA ST398 strains, as assessed by a log rank test (*p* < 0.05 after Bonferroni correction for multiple testing. *n* = 10/group). **(B)** Cohorts of BALB/c mice (*n* = 8–10) were treated by injection into the retroorbital plexus with either 1 × 10^8^ CFU of *S. aureus* ST239 or the Δ*spa* mutant. On day 7 or 15 postchallenge, animals were killed to enumerate staphylococcal burden in renal tissues. No mortality was observed during the sublethal challenge. **(C-E**′**)** Representative hematoxylin and eosin stained histopathology slides of the kidney on day 15 postchallenge. White arrows indicate staphylococcal abscess communities. Histopathology images were acquired with light microscopy at × 40 **(C–E)** and × 200 **(C**′**–E**′**)** magnification. Scale bars represent 100 μm **(C–E)** and 25 μm **(C**′**–E**′**)** respectively. Animal data are representative of 3 independent experiments. ^*^p < 0.05, ^***^p < 0.001.

SpA contributes to *S. aureus* virulence in infections such as arthritis and sepsis (Palmqvist et al., 2002). Resistance to methicillin of HA-MRSA affects bacteria's *agr* sensing system, leading to reduced virulence and inability to move into the community, while the emergent community-associated MRSA (CA-MRSA) typically express less penicillin-binding protein 2a (encoded by mecA), allowing them to maintain full virulence and succeed in the community environment (Rudkin et al., 2012). In order to test the infection mortality between the epidemic HA-MRSA ST239 of high SpA expression and CA-SA ST398, an acute sepsis model was developed in BALB/c mice. Groups of mice were inoculated intravenously with clinical ST239 or ST398 isolates. The ST398 strains gave rise to a significantly higher (*p* < 0.001) mortality rate than ST239 (Figure 7A). The mortality of ST239 was relatively lower than ST398.

The CA-SA ST398 could produce multitudes of external toxins, enhancing its invasive ability and easily causing the more severe infections in healthy individuals, while ST239 produce higher amounts of protein A. To explore the higher expression of protein A in the pathogenicity of ST239 infections, we then developed a mouse renal abscess model with sublethal challenged bacteria WT Ji99 and its isogenic *spa* mutant strain, and evaluated the severity of infection by enumerating staphylococcal CFU per kidney. Both 7 and 15 days after inoculation, the staphylococcal loads per kidney were decreased in the Δ*spa* mutant-infected mice compared with the WT-infected mice (Figure 7B). Tissue damage in development of abscess formation in kidneys of the mutant-infected mice was also alleviated contrast to the WT-infected mice (Figures 7C′–E′). These data suggested that protein A was a prominent factor working in abscess lesions, contributing largely to the durative damage with HA-MRSA ST239 infection.

## Discussion

*S. aureus* is a common pathogen contributing to multitudes of infections. MRSA, responsible for innumerable drug-resistant healthcare-associated infections globally, has evolved with progressive adaptation to its environment. Recent studies have demonstrated the crucial adaptive responses of HA-MRSA to the highly selective environment of the healthcare system, and the evolution of HA-MRSA isolates to even higher levels of antibiotic resistance at the cost of attenuated virulence (Baines et al., 2015), which improves its ability to become a successful nosocomial pathogen. High expression level of mecA in HA-MRSA affects bacteria's *agr* sensing system, leading to reduced virulence and inability to move into the community, while the emergent CA-MRSA typically express less penicillin-binding protein 2a (encoded by mecA), allowing them to maintain full virulence and succeed in the community environment (Rudkin et al., 2012). HA-MRSA strains are less virulent as a direct consequence of expressing the *mecA* gene at high levels (Rudkin et al., 2012) in addition to the expression of *psm-mec*, a putative virulence regulatory locus as well as a cytolytic phenol-soluble modulin having been identified on the healthcare-associated type II SCC*mec* element (Queck et al., 2009; Kaito et al., 2011).

In our study, we focused on the global spread HA-MRSA ST239, the predominant hospital infection in China for decades and quite distinct from the emerging CA-SA ST398 infecting healthy populations with no contact to healthcare settings and no association with animals (Zhao et al., 2012). To investigate what enables HA-MRSA ST239 to successfully colonize in a hospital setting and gradually progress to a series of widespread invasive infections, we searched for additional contributing factors, other than the existent expression of *psm-mec* and *mecA* that attenuate virulence and promote its spread in nosocomial settings. We discovered higher expression of protein A by HA-MRSA ST239 compared with CA-SA ST398. Notably, protein A, a highly conserved protein expressed by all *S. aureus* isolates, is an important immunomodulatory factor blocking opsonophagocytosis through the Fc ɤ-binding domain and inducing B cell apoptosis by binding to the Fab domains and crosslinking of V_H_3 clan IgM in B cells. To a large extent, SpA-neutralizing antibodies cannot be developed during infections. The superantigen activity of SpA leads to immunodominance, limiting host responses to other *S. aureus* virulence factors (Pauli et al., 2014). SpA may constitute a crucial determinant in the colonization and spread of the epidemic HA-MRSA ST239 clone in hospital settings.

Protein A, a cell wall-anchored protein, can also be released into the extracellular surroundings to access the host's immune system (Becker et al., 2014). *spa* is expressed by all clinical *S. aureus* isolates with conserved immunoglobulin binding domains. However, its expression in different clones may be of much diversity as shown in our study. SpA is also detectable at the RNA level in nasal swabs (Burian et al., 2010). The exoproteome of a nasal carrier strain of *S. aureus* was compared to a genetically similar non-carrier strain, revealing that Staphylococcal protein A was present at significantly higher levels in carrier strains than the non-carrier, which suggests an association of protein A with nasal carriage (Muthukrishnan et al., 2011). We observed that increased expression of protein A in HA-MRSA ST239 enhanced nasal colonization of this clone. The mechanism behind the colonization promotion may be the sequentially glycosylation of SpA in nasal carrier strains, while glycosylation of exoproteins such as SpA might play crucial roles in bacterial pathogenesis and immunoevasion (Schmidt et al., 2003; Muthukrishnan et al., 2011). Other than nasal cavity, *S. aureus* colonize in skin, armpits and crissum. The colonization may be associated with the interaction between SpA and epidermal keratinocytes. There are studies discovering the important role of protein A for the staphylococcal adhesion to human epidermal cells (Cole and Silverberg, 1986; Mempel et al., 1998). This was confirmed by the low adhesion capacity of protein A-negative mutant Ji99Δ*spa* and 1059Δ*spa* strains to the cultured monolayers. Its counterpart on human keratinocytes may be cytoskeletal β-actin. The binding of SpA to mammalian epithelial actin filaments plays a role in the internalization and dissemination of *S. aureus* (Jung et al., 2001). We then tested this binding affinity of SpA in the human epithelial cells, which is involved in the regulation of *S. aureus* invasion by epithelial cells with the elongation of actin filaments by their polymerization. Here we see that the increased SpA of ST239 enhanced the adhesion to keratinocytes, as well as epithelial cells. And it helps to promote the colonization of ST239 in human in the way of positive feedback. Additionally, the two *spa* deletion strains of ST239 and ST398 were seen statistically different in colonization and adhesion, indicating that there are other factors involved in this process except protein A.

Risk factors for *S. aureus* infection include recent surgery, admission to a hospital or nursing home, antibiotic use, dialysis, and permanent indwelling catheter (Nathwani et al., 2008). Upon entering the host, HA-MRSA ST239 defends against the immune response by early resisting neutrophils' phagocytosis and inducing activated-B cell apoptosis through the expression of surface-anchored and released SpA, suppressing the production of neutralizing antibodies against SpA and many other antigens, which facilitates its dissemination from sites of infection to other regions.

Previous studies have demonstrated that endogenous TNF plays an important role in host resistance to *S. aureus* infection, while endogenous IFN-ɤ provides protection in the early stage of infection and plays a detrimental role late in infection (Nakane et al., 1995). TNF has been shown to alter many properties of neutrophils *in vitro*: enhancement of phagocytosis, induction of degranulation, stimulation of adherence, and stimulation of the generation of superoxides. Mice lacking capacity to produce TNF-α have higher mortality and inefficient bacterial clearance in *S. aureus* arthritis and brain abscess model (Hultgren et al., 1998; Kielian et al., 2004). Protein A recognizes TNFR1 on epithelial cells and induces inflammation through TNFR1 in the way of TNF-α-TNFR1 signaling activation, leading to recruitment and activation of neutrophils and bacterial clearance from the airway, though at the cost of PMN-associated epithelial damage and respiratory compromise (Gomez et al., 2004). Protein A plays another key role in inducing early shedding of TNFR1 on macrophages. The increased levels of soluble TNFR1 present during systematic HA-MRSA ST239 infection may neutralize circulating TNF-α and impair the host inflammatory response (Giai et al., 2013). The combination of anti-TNF-α drugs and antibiotic alleviates the outcome of *S. aureus* arthritis and sepsis in a mouse model (Fei et al., 2011; Ali et al., 2015). The action of soluble TNFR1 much resembles the anti-TNF-α drugs, suggesting that blocking TNF-α indeed increases bacterial survival in infections and benefits the host by decreasing the damage caused by inflammation. Perhaps that also partially explains why ST239 seem to be less virulent and can colonize hosts for a longer time. It is a novel strategy for ST239 subverting the host immune response. The TNFα-TNFR1 regulation is more complicated after *S. aureus* infection *in vivo*, depending largely on the type and stage of diseases, and differs in localized and systemic infections (Giai et al., 2013). Then we need to do more experiments to elucidate the process in our following studies.

There is a difference in *spa* gene structure between ST239 and ST398 clones: the length of *spa* gene in ST239 is shorter than that of ST398, which mainly exists in Domain D, Domain A and Xr region. Does the difference between the two clinical strains have connection with their different *spa* gene structures, other than different levels of protein A production? To address the question, we constructed the two *spa* complemented strains: Ji99Δ*spa*(p239) complemented with the ST239 *spa* gene and 1059Δ*spa*(p398) with ST398 *spa* gene. The Western Blot data showed that the expression of protein A in Ji99Δ*spa*(p239) and 1059Δ*spa*(p398) were almost in the same level (Figure 4A). Furthermore, the two *spa* complemented strains can reverse the phenotypes, including the nasal colonization in mice, the adhesion ability to cells, anti-phagocytosis and TNFR1 shedding, to the wild type level (Figures 3, 4B, 5C, 6B–D), suggesting that the difference is not due to the distinct *spa* structures between ST239 and ST398 but the expression of this protein.

Protein A also acts as a virulence factor in *S. aureus* infections and is necessary for the development of abscess formation (Palmqvist et al., 2002; Cheng et al., 2009). Palmqvist et al. reported the elevated levels of TNF-α in infections is caused by protein A-deficient mutants, which is in consistent with our results about SpA shedding of TNFR1 on macrophages. In the acute sepsis model (with high bacterial load: 1 × 10^9^ CFU), the mortality caused by ST239 was significantly lower than ST398 (Figure 7A), suggesting that SpA may contribute to the low mortality in ST239 infections with higher SpA expression rather than the acute infection caused by CA-ST398, while the production of a plethora of external toxins in ST398 may enhance its pathogenicity. According to the report of Cheng et al. SpA is necessary for the development of abscess formation. We then tested the function of SpA by using sublethal dose of bacterial infection (with low bacterial load: 1 × 10^8^ CFU) challenged with ST239, along with its isogenic *spa* mutant strain, and observed its role in tissue abscess formation. Our data suggested that SpA exactly promoted the durative damage in renal, as the isogenic *spa* mutant strain of ST239 had relatively lower bacterial load and alleviated tissue damage in kidney (Figures 7B–E′). All of above data suggest that SpA is not a key virulence factor in acute infections of *S. aureus*, it may contribute to the durative damage in host infected with HA-MRSA ST239.

Protein A is an exceptional virulence factor, a single protein that can target multiple immunologically important eukaryotic receptors. Probably, it is not a coincidence that protein A is amongst the most highly conserved staphylococcal virulence factors expressed, nor that its levels of expression are significantly increased in staphylococci isolated from invasive human infections (Gomez et al., 2007). To summarize, we suggest that protein A may serve as a crucial determinant in the colonization and immune evasion of HA-MRSA ST239 infections, thus contributing to persistent spread in the hospital settings.

The highly successful adaptive HA-MRSA ST239 is a growing concern worldwide. The combination of anti-TNF-α drugs and antibiotic may have little use in the treatment therapy against ST239 infections. Researchers have focused on immunization with non-toxigenic protein A (SpA_KKAA_) or administration of protein A-neutralizing monoclonal antibodies, thus eliciting protective antibodies in mice against highly virulent MRSA strains (Kim et al., 2010, 2012). Furthermore, it has been proposed that SpA_KKAA_ represents a protective antigen for the development of a staphylococcal vaccine (Falugi et al., 2013). We hope that the vaccine could inform novel prevention policies in health care settings, and the development of new antibodies to treat HA-MRSA infections.

## Author contributions

ML and YL conceived the study. XH, JQ, TL,YD, YW, and QL performed experiments. XH, TL, LH, and ML analyzed data. XH and ML drafted the manuscript. XH, JQ, HL, QG, YL, and ML revised and approved the manuscript.

### Conflict of interest statement

The authors declare that the research was conducted in the absence of any commercial or financial relationships that could be construed as a potential conflict of interest.

## Supplementary material

Supplementary Figure 1 **The original gels concerning the anti-SpA western blot of different clinical isolates in ST239 and ST398**. The number of ST398 isolates: 1–14. The number of ST239 isolates: 15–28.

Supplementary Table 1 **Bacterial strains and plasmids used in this study**.

Supplementary Table 2 **Oligonucleotides used in this study**.
