# Supplementary material for: Staphylococcal Protein A Promotes Colonization and Immune Evasion of the Epidemic Healthcare-Associated MRSA ST239
Source: Front Microbiol. 2016 Jun 27;7:951. doi: 10.3389/fmicb.2016.00951 (PMC4922140; doi:10.3389/fmicb.2016.00951)
Supplement: Supplementary Figure 1 — The original gels concerning the anti-SpA western blot of different clinical isolates in ST239 and ST398. The number of ST398 isolates: 1–14. The number of ST239 isolates: 15–28. [file Table1.pdf]

Supplementary Materials

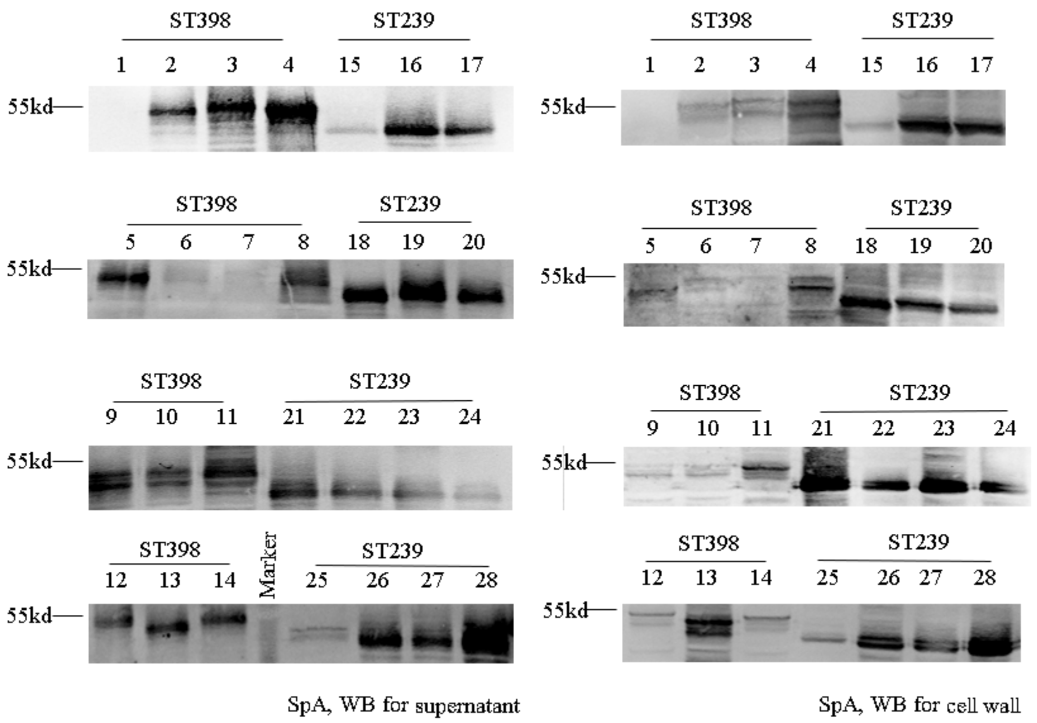

Supplementary Figure. The original gels concerning the anti-SpA western blot of different clinical isolates in ST239 and ST398.
The number of ST398 isolates: 1-14
The number of ST239 isolates: 15-28

Supplementary Table 1. Bacterial strains and plasmids used in this study.

**Strain / plasmid**	**Relevant genotype and property**	**Source / reference**
S. aureus		
RN4220	Derived from NCTC8325-4;r-m+	(Kreiswirth *et al.*, 1983)
ST239-Ji99	HA-MRSA clinical isolate	This study
Ji99Δ*spa*	Ji99Δ*spa* mutant	This study
ST398-1059	CA-MRSA clinical isolate	This study
1059Δ*spa*	1059Δ*spa* mutant	This study
Ji99Δ*spa* (pCL55)	Ji99Δ*spa* mutant with pCL55	This study
Ji99Δ*spa* (p239)	Ji99Δ*spa* mutant with pCL55 carrying Ji99 *spa* gene	This study
Ji99Δ*spa* (p398)	Ji99Δ*spa* mutant with	This study

	pCL55 carrying 1059 *spa* gene	
E. coli		
DH5α	*endA*1 *recA*1 *gyrA*96 *thi-1 hsdR17*(rK- mK+) *relA*1 *supE*44 (*lacZYA-argF*)U169 F-80d*lac*ZM15 *deoR phoA*	Invitrogen
Plasmids		
pKOR1	cmR and ampR, temperature-sensitive vector for allelic replacement via lambda recombination and *ccdB* selection	(Bae & Schneewind, 2006)
pKOR1 *spa*	Vector for allelic replacement of *spa* in *S. aureus*	This study
pCL55	*E. coli/Staphylococcus* shuttle cloning plasmid, *cm*R	(Lee *et al.*, 1991)
pCL55*spa*	pCL55 with insertion of *spa* gene	This study

Supplementary Table 2. Oligonucleotides used in this study.

Oligonucleotide	Sequence
Oligonucleotides for isogenic deletion mutants	
spa-att1	GGGGACAAGTTTGTACAAAAAAGCAGGCT GATGGCGACAGACAAAACCAAGC
spa-rev1	ATTAATACCCCCTGTATGTATTTG
spa-rev2	CAAATACATACAGGGGGTATTAATCTTAT ATACGTTGATTAACACATTC
spa-att2	GGGGACCACTTTGTACAAGAAAGCTGGGT GTGCCCATTTCAATGTTTCAAC
Oligonucleotides for genetic complementation	
spa-Sma1-F	GAGCCCGGGCGCAAGTGTGCTGT
spa-BamH1-R	GAGGGATCCTTATAGTTCGCGACGACGTC
Oligonucleotides for qRT- PCR	

gyrB-F	CAAATGATCACAGCATTTGGTACAG
gyrB-R	CGGCATCAGTCATAATGACGAT
spa-F	CAGCAAACCATGCAGATGCTA
spa-R	ACCGATGAATGGATTTTCTTCAC
hla-F	AATAACTGTAGCGAAGTCTGGTGAAA
hla-R	GCAGCAGATAACTTCCTTGATCCT
Oligonucleotides for spa typing	
T*spa*-F	TAAAGACGATCCTTCGGTGAGC
T*spa*-R	CAGCAGTAGTGCCGTTTGCTT
